# Brain metabolic sensing and metabolic signaling at the level of an astrocyte

**DOI:** 10.1002/glia.23283

**Published:** 2017-12-23

**Authors:** Nephtali Marina, Egor Turovsky, Isabel N Christie, Patrick S Hosford, Anna Hadjihambi, Alla Korsak, Richard Ang, Svetlana Mastitskaya, Shahriar Sheikhbahaei, Shefeeq M Theparambil, Alexander V Gourine

**Affiliations:** ^1^ Centre for Cardiovascular and Metabolic Neuroscience, Neuroscience, Physiology & Pharmacology University College London London WC1E 6BT United Kingdom; ^2^ Research Department of Metabolism and Experimental Therapeutics, Division of Medicine University College London London WC1E 6JJ United Kingdom; ^3^ Laboratory of Intracellular Signalling Institute of Cell Biophysics, Russian Academy of Sciences Pushchino Russia

**Keywords:** brainstem, breathing, chemoreception, food intake, gut hormone, metabolism

## Abstract

Astrocytes support neuronal function by providing essential structural and nutritional support, neurotransmitter trafficking and recycling and may also contribute to brain information processing. In this article we review published results and report new data suggesting that astrocytes function as versatile metabolic sensors of central nervous system (CNS) milieu and play an important role in the maintenance of brain metabolic homeostasis. We discuss anatomical and functional features of astrocytes that allow them to detect and respond to changes in the brain parenchymal levels of metabolic substrates (oxygen and glucose), and metabolic waste products (carbon dioxide). We report data suggesting that astrocytes are also sensitive to circulating endocrine signals—hormones like ghrelin, glucagon‐like peptide‐1 and leptin, that have a major impact on the CNS mechanisms controlling food intake and energy balance. We discuss signaling mechanisms that mediate communication between astrocytes and neurons and consider how these mechanisms are recruited by astrocytes activated in response to various metabolic challenges. We review experimental data suggesting that astrocytes modulate the activities of the respiratory and autonomic neuronal networks that ensure adaptive changes in breathing and sympathetic drive in order to support the physiological and behavioral demands of the organism in ever‐changing environmental conditions. Finally, we discuss evidence suggesting that altered astroglial function may contribute to the pathogenesis of disparate neurological, respiratory and cardiovascular disorders such as Rett syndrome and systemic arterial hypertension.

## Main Points

- Astrocytes are versatile CNS sensors capable of detecting various metabolic and endocrine signals.
- Astrocytes modulate the activities of the brainstem respiratory and autonomic neuronal circuits that ensure metabolic homeostasis.

## 1 INTRODUCTION

Living cells generate a constant supply of adenosine triphosphate (ATP) to provide the energy required to carry out fundamental cellular processes, such as cytoskeleton assembly, maintenance of membrane potential and excitability, membrane transport, cell movement/migration, intracellular and intercellular signaling. A complex hierarchy of behavioral, physiological and biochemical mechanisms ensure adequate delivery of metabolic substrates and effective elimination of metabolic waste products from all tissues of the body (Fell, 1997).

The central nervous system (CNS) plays a key role in the maintenance of energy homeostasis. This function requires specific sensors that can rapidly respond to perturbations in the metabolic milieu. A series of seminal studies identified groups of neuronal metabolic sensors located in discrete brain areas, particularly in the hypothalamus and the brainstem (Anand, Chhina, Sharma, Dua, & Singh, 1964; Oomura et al., 1964; Oomura, Nakamura, Sugimori, & Yamada, 1975; Loeschcke, 1982). Like all brain cells, these neurons use metabolic substrates to satisfy their own metabolic needs, but in addition, they also have the ability to respond to variations in the availability of specific substrates with changes in membrane excitability, neurotransmitter release and gene expression. This, in turn, results in adaptive physiological responses that control multiple aspects of energy balance such as oxygen delivery, carbon dioxide elimination and feeding behavior, among others. More recently it has become evident that CNS metabolic sensing is more complex than originally thought and may involve glial cells in an active role.

In this special issue of *GLIA* we discuss the emerging evidence supporting the hypothesis that astrocytes function as versatile metabolic sensors of CNS milieu and by doing so play an important role in the maintenance of brain metabolic homeostasis. Cellular features of astrocytes allow them to detect and respond to changes in the brain parenchymal levels of metabolic substrates and metabolic waste products. Astrocytes are also sensitive to circulating hormones that modulate the activities of the neuronal circuits controlling food intake and energy balance. Recent evidence suggests that astrocytes modulate the activities of vital respiratory and autonomic neuronal networks that control breathing and autonomic balance while compromised astroglial function may contribute to the development and progression of disparate neurological, respiratory and cardiovascular diseases.

## 2 ASTROCYTES

Astrocytes support neuronal function by providing structural and nutritional support as well as by facilitating neurotransmitter trafficking and recycling. There is also significant evidence to suggest that astrocytes contribute to CNS information processing (Halassa et al., 2009; Papouin, Dunphy, Tolman, Foley, & Haydon, 2017). The morphological and functional adaptations of astrocytes ideally position them to act as physiological sensors of brain metabolic milieu: (i) Sensory input: perturbations in metabolic milieu as well as systemic hormonal signals are detected by astrocytes residing in the hypothalamus and the brainstem (Chowen et al., 1999; Cheunsuang & Morris, 2005; Angelova et al., 2015; Garcia‐Caceres et al., 2016; Turovsky et al., 2016). Astroglial processes and end feet surrounding the cerebral vasculature form one of the key elements of the blood–brain barrier. As a result, astrocytes are ideally placed to sense blood‐borne metabolic and endocrine signals (Kacem, Lacombe, Seylaz, & Bonvento, 1998; Sofroniew & Vinters, 2010); (ii) Transduction mechanisms: astrocytes are not electrically excitable but display so‐called “Ca^2+^ excitability” responding to various stimuli (e.g., chemical, mechanical, etc.) and certain neuronal cues with increases in intracellular [Ca^2+^] (Zheng et al., 2015; Bazargani & Attwell, 2016) followed by intracellular changes and/or the release of various signaling molecules (“gliotransmitters”). (iii) Neuromodulatory output: astrocytes have a dense network of finely branching processes that enwrap neuronal synapses forming one of the components of the so‐called “tripartite synapse” (Perea, Navarrete, & Araque, 2009). These processes contain membrane proteins that play important roles in ensuring effective synaptic transmission such as glutamate transporters (Chaudhry et al., 1995), potassium channels (Higashi et al., 2001; Olsen, 2012), aquaporins (Thrane et al., 2011), and lactate transporters (Puchades, Sogn, Maehlen, Bergersen, & Gundersen, 2013). Increases in intracellular [Ca^2+^] in astrocytes can also trigger the release of gliotransmitters that interact with pre‐ and post‐synaptic receptors and can potentially control neuronal network activity via modulation of synaptic transmission and neuronal excitability (Perea et al., 2009). Several molecules have been suggested to function as gliotransmitters, including ATP/adenosine, polyphosphate, d‐serine, glutamate, GABA, and lactate (Volterra & Meldolesi, 2005; Rollenhagen et al., 2007; Holmstrom et al., 2013; Tang et al., 2014; Marina et al., 2015; Martin, Bajo‐Graneras, Moratalla, Perea, & Araque, 2015; Papouin et al., 2017).

Thus, astrocytes appear to be strategically positioned to monitor the chemical composition of the arterial blood entering the brain, integrate it with the metabolic signals arising from the brain parenchyma and communicate this information to intermingled neuronal networks, enabling the initiation of coordinated adaptive physiological and behavioral responses that ensure homeostasis in dynamic environmental conditions (Gourine, 2005; Gourine & Kasparov, 2011; Teschemacher, Gourine, & Kasparov, 2015). Astrocytes are also at the center of the neurovascular interface and are able to release vasoactive molecules that regulate cerebral blood flow in accord with prevailing neuronal activity. This facilitates the supply of oxygen and glucose and the removal of CO_2_ in a process known as neurovascular coupling (Attwell et al., 2010).

## 3 ASTROCYTES AS CNS METABOLIC SENSORS

### 3.1 Sensing oxygen

Aerobic respiration is the key cellular process which breaks down metabolic substrates to produce molecules of ATP. In air‐breathing animals the supply of oxygen and the removal of carbon dioxide involve the transfer of air between the atmosphere and the lungs by the process of alveolar ventilation, the diffusion of gas between alveoli and the pulmonary blood and the transport of oxygen and carbon dioxide to and from all tissues of the body, respectively. The partial pressure of oxygen (*P*O_2_) in the arterial blood is sensed by the peripheral oxygen chemoreceptors located in the carotid bifurcation and in the aortic arch (in some species). The chemosensitive glomus cells of the carotid body are traditionally considered to be the primary (and only) respiratory oxygen sensors in mammals. When activated by hypoxia, carotid bodies initiate a chemoreflex that results in activation of the respiratory and sympathetic circuits located in the brainstem. This leads to rapid respiratory and cardiovascular responses directed towards restoring the arterial *P*O_2_ (Guyenet, 2006; Kumar & Prabhakar, 2012). However, there is significant evidence that all mammals can survive denervation of the peripheral oxygen sensors and that hypoxic ventilatory response recovers in experimental animals whose carotid bodies have been surgically denervated or removed, suggesting that the brain may also contain functional respiratory oxygen sensors (Davenport, Brewer, Chambers, & Goldschmidt, 1947; Miller & Tenney, 1975; Olson, Vidruk, & Dempsey, 1988; also see Gourine & Funk, 2017 for a comprehensive review).

**Figure 1:**
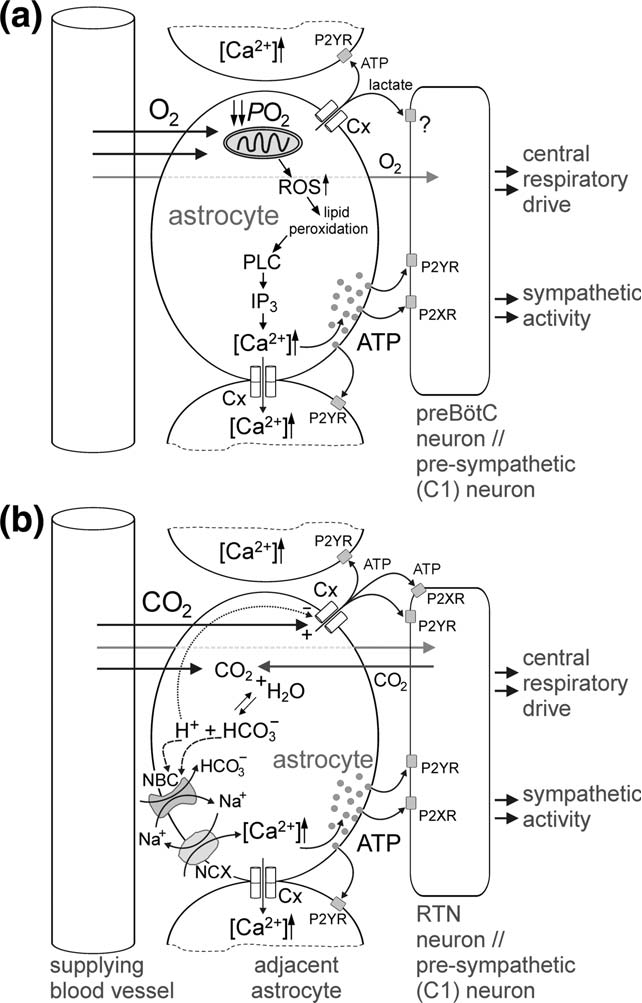
Hypothesized cellular mechanisms underlying astroglial oxygen and CO_2_/pH sensitivities. (a) The astroglial signaling cascade triggered by hypoxia involves inhibition of mitochondrial respiration, facilitated formation of reactive oxygen species (ROS), lipid peroxidation, activation of phospholipase C (PLC), IP_3_ receptors, release of Ca^2+^ from the intracellular stores and enhanced vesicular release of ATP. Hypoxia may also alter opening probability of connexin (Cx) hemichannels permeable to ATP and lactate. (b) Increases in *P*CO_2_ gate open Cx hemichannels in Ca^2+^ and pH‐independent manner allowing rapid egress of ATP in response to hypercapnia. If hypercapnic stimulus is persistent, intracellular acidification will occur and will eventually close Cx hemichannels. In conditions of decreased pH_i_, astrocytes continue to release ATP by Ca^2+^‐dependent exocytotic release mechanism. Intracellular acidification activates Na^+^/ ${\text{HCO}}_{3}^{-}$ cotransport (NBC) which brings Na^+^ inside the cell. Raising [Na^+^]_i_ activates Na^+^/Ca^2+^ exchanger (NCX) to operate in a reverse mode leading to Ca^2+^ entry. Released ATP acting in autocrine and paracrine manner spreads astroglial Ca^2+^ signals within the neuropil and enhances respiratory and sympathetic activities via excitation of the respiratory rhythm generating circuits of the pre‐Bötzinger complex (preBötC), retrotrapezoid nucleus (RTN) neurons and sympathoexcitatory (pre‐sympathetic) neurons of the brainstem

**Figure 2:**
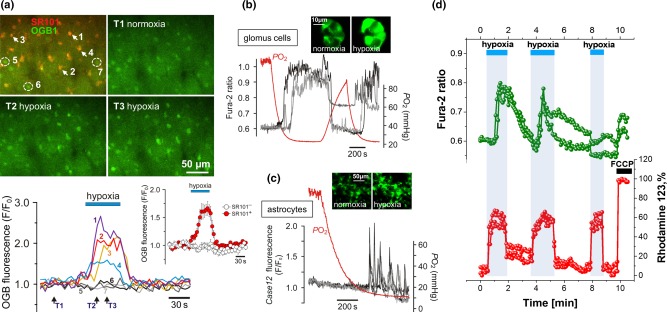
Astrocytes are sensitive to physiological decreases in *P*O_2_. (a) *In vivo* imaging of hypoxia‐evoked astrocytic [Ca^2+^]_i_ responses in somatosensory cortex of an anesthetized adult rat. Top: pseudocolored images showing changes in oregon green BAPTA 1 (OGB1) fluorescence taken at times indicated by arrows on the bottom panel. 5–7—non‐responding cells that were not labeled with sulforhodamine 101 (SR101). Bottom: traces showing changes in astrocytic [Ca^2+^]_i_ in response to hypoxia. Inset: averaged changes in OGB1 fluorescence induced by hypoxia in ten SR101 labeled cells (SR101^+^, putative astrocytes) and five neighboring cortical cells that lacked SR101 labeling (SR101^−^, likely neurons) recorded in this experiment; (b) hypoxia‐induced [Ca^2+^]_i_ responses of carotid body glomus cells in culture, visualized using Ca^2+^ indicator Fura‐2 (*P*O_2_ threshold of activation 40 mmHg). Inset: pseudocolored images of a cluster of glomus cells showing changes in Fura‐2 fluorescence in response to hypoxia (hypoxic conditions *in vitro* were induced by gradual displacement of oxygen in the incubation medium with argon); (c) hypoxia‐induced [Ca^2+^]_i_ responses of brainstem astrocytes (*P*O_2_ threshold of activation 15 mmHg). In this example astrocytes were identified and their responses to hypoxia were assessed in organotypic brainstem slice *in vitro* using genetically encoded Ca^2+^ sensor *Case12* expressed under the control of GFAP promoter; (d) simultaneous imaging of hypoxia‐induced changes in Δψm and [Ca^2+^]_i_ in cultured brainstem astrocytes using Rhodamine 123 and Fura‐2 showing that mitochondrial depolarization precedes Ca^2+^ responses. Mitochondrial depolarization is induced by FCCP (1 µM) applied at the end of the experiment to calibrate the Rh123 signal (100%). Reproduced from Angelova et al. (2015) with permission from the Society for Neuroscience

Results of recent studies suggested that astrocytes may function as physiological sensors of brain oxygenation (Figure 1; Angelova et al., 2015). This was demonstrated using two‐photon imaging of cortical astrocytes in anesthetized and mechanically ventilated rats, where decreases in inspired O_2_ (from 21% to 15% or 10%) were found to trigger robust increases in astroglial [Ca^2+^]_i_ (Angelova et al., 2015; Figure 2). *In vitro* experiments revealed that oxygen sensitivity is a general feature of brain astrocytes and that the hypoxia sensor is located in the mitochondria. Simultaneous measurements of mitochondrial membrane potential (Δψ_m_) and [Ca^2+^]_i_ in cultured astrocytes showed that a decrease in *P*O_2_ causes a significant decrease in Δψ_m_ and that this response precedes increases in [Ca^2+^]_i_ (Figure 2d). Inhibition of mitochondrial respiration in these conditions was accompanied by increases in mitochondrial reactive oxygen species (ROS) production. Both hypoxia‐induced ROS production and [Ca^2+^]_i_ responses in astrocytes were markedly reduced or abolished by mitochondrial uncoupler (FCCP), mitochondrial antioxidant (MitoQ) or ROS scavenger (α‐tocopherol). Subsequent pharmacological analysis of Ca^2+^ responses suggested a feasible hypoxia‐sensitive signaling pathway: in astrocytes hypoxia leads to inhibition of mitochondrial respiration, increased production of free radicals, lipid peroxidation, activation of phospholipase C and recruitment of Ca^2+^ from IP_3_‐sensitive intracellular stores (Figure 1) (Angelova et al., 2015).

It was reported that the *P*O_2_ hypoxia threshold required to trigger Ca^2+^ responses in astrocytes is ∼17 mmHg (Angelova et al., 2015). Normal level of arterial *P*O_2_ is ∼100 mmHg. Normal level of brain parenchymal *P*O_2_ is between 20 and 30 mmHg with little difference between the mammalian species (Erecińska & Silver, 2001). A study conducted in climbers of Mt Everest reported mean arterial *P*O_2_ of ∼25 mmHg in four individuals breathing ambient air at 8400 m above sea level (Grocott et al., 2009). Although we do not know what was the brain tissue *P*O_2_ in these conditions, these fascinating data suggest that the brain can operate in a very low oxygen environment (the participants were able to perform complex tasks), with parenchymal *P*O_2_ sufficiently low to trigger astroglial activation and downstream sympathetic, respiratory and regional cerebrovascular responses (see below).

Experiments conducted to visualize vesicular fusion events in cultured rat brainstem astrocytes using total internal reflection fluorescence microscopy demonstrated that reductions in *P*O_2_ facilitate exocytosis of ATP‐containing vesicles (Angelova et al., 2015; Figure 1). Earlier *in vitro* and *in vivo* experiments using amperometric enzymatic ATP biosensors (Gourine et al., 2007; Gourine et al., 2008) showed that brain tissue hypoxia is indeed associated with the release of ATP, specifically in the brainstem areas which harbor the respiratory and sympathetic neuronal circuits (Gourine, Llaudet, Dale, & Spyer, 2005). Activation of these networks by ATP triggers compensatory cardiorespiratory responses that facilitate central respiratory drive, alveolar ventilation and oxygen transport.

The rhythm and pattern of breathing are generated by complex neuronal interactions within the so‐called brainstem ventral respiratory column that extends from the dorsolateral pons to the caudal regions of the medulla oblongata (Feldman, Mitchell, & Nattie, 2003; Feldman, Del Negro, & Gray, 2013). The brainstem respiratory network ensures that the frequency, depth and pattern of lung ventilation are always adequate to maintain blood and brain *P*O_2_, *P*CO_2_ and pH within physiological ranges. Neurons that constitute the brainstem respiratory‐rhythm generating circuits express ATP receptors and are strongly excited by ATP (Thomas & Spyer, 2000; Gourine, Atkinson, Deuchars, & Spyer, 2003; Huxtable et al., 2009). Recent data demonstrated that ATP, released by brainstem astrocytes in response to hypoxia, contributes to the development of the hypoxic ventilatory response (Angelova et al., 2015; Rajani et al., 2017). In rats, blockade of astroglial signaling in the ventral regions of the brainstem by overexpression of ATP‐degrading enzymes or targeted astrocyte‐specific expression of tetanus toxin light chain (to block vesicular release mechanisms) was found to be associated with a significant reduction of the hypoxic ventilatory response. These data strongly suggested that the central stimulatory effect of hypoxia on breathing is mediated by an astroglial purinergic signaling mechanism (Angelova et al., 2015; Rajani et al., 2017).

The hypoxic ventilatory response is also accompanied by a coordinated cardiovascular response to ensure effective delivery of the oxygenated blood to all tissues of the body. Oxygen delivery is determined by cardiac output that is controlled (among other factors) by the activities of the sympathetic and parasympathetic branches of the autonomic nervous system. Groups of sympathoexcitatory (pre‐sympathetic) brainstem neurons, including bilateral populations of catecholaminergic C1 neurons, are essential for the generation of cardiovascular sympathetic tone (Ross et al., 1984; Reis, Golanov, Ruggiero, & Sun, 1994; Guyenet, 2006; Marina et al., 2011; Wenker et al., 2017). These neurons send monosynaptic projections to sympathetic preganglionic neurons in the intermediolateral spinal cord, which in turn project to sympathetic ganglia innervating peripheral targets, including the heart, kidneys and the resistance arterioles of the skeletal muscle (Guyenet, 2006; Guyenet et al., 2013). There is strong evidence that the activities of the brainstem pre‐sympathetic neuronal circuits are modulated by astrocytes (Marina et al., 2013; Marina et al., 2015). It was reported that [Ca^2+^]_i_ responses in astrocytes induced by optogenetic stimulation lead to excitation of neighboring C1 neurons in the brainstem slices *in vitro* and trigger increases in renal sympathetic nerve activity, heart rate and the systemic arterial blood pressure in anesthetized rats *in vivo* (Marina et al., 2013).

The physiological significance of astroglial control of pre‐sympathetic brainstem neurons becomes apparent during central hypoxia. These sympathoexcitatory neurons are known to be highly sensitive to decreases in local tissue *P*O_2_ or cytotoxic hypoxia, responding with profound excitation leading to generalized increases in central sympathetic drive (Sun & Reis, 1994; D'Agostino, Mazza, & Neubauer, 2009; Marina et al., 2015). It appears that the sensitivity of C1 pre‐sympathetic neurons to hypoxia is largely indirect, and mediated by the actions of ATP and lactate released by neighboring astrocytes (Figure 1). First, hypoxia‐induced excitation of C1 neurons was found to be markedly reduced by blockade of either metabotropic ATP receptors or inhibition of glycogenolysis (Marina et al., 2015). Second, excitation of C1 neurons following optogenetic stimulation of astrocytes was significantly reduced in the presence of an ATP‐degrading enzyme apyrase (Marina et al., 2013). Third, exogenous ATP and l‐lactate induced potent excitation of C1 neurons *in vitro* and triggered sympathoexcitatory effects similar to those observed following optogenetic activation of brainstem astrocytes or brain hypoxia *in vivo* (Sun, Wahlestedt, & Reis, 1992; Horiuchi, Potts, Tagawa, & Dampney, 1999; Ralevic, 2000; Marina et al., 2015). Finally, hypoxia facilitated release of both ATP (Gourine et al., 2005) and lactate (Karagiannis et al., 2016; Hadjihambi et al., 2017) in brainstem regions containing populations of pre‐sympathetic neurons. While the effect of ATP on C1 neuronal activity is mediated by P2X and P2Y receptors (Sun et al., 1992; Ralevic, 2000; Wenker, Sobrinho, Takakura, Mulkey, & Moreira, 2013), the mechanisms underlying the excitatory effects of lactate remain unknown but appear to involve activation of as yet uncharacterized lactate‐sensitive G_s_ coupled receptor (Tang et al., 2014).

The experimental data reviewed and discussed above suggest that astrocytes are intrinsically sensitive to hypoxia and play an important role in the development of coordinated ventilatory and cardiovascular responses to decreases in brain (stem) *P*O_2_. There is also evidence that astroglial oxygen sensitivity may contribute to the pathogenesis of certain diseases associated with brain hypoxia. Congestive heart failure and systemic arterial hypertension are highly prevalent conditions characterized by sustained increases in sympathetic nerve activity, which is generally believed to have long‐term detrimental effects and contribute to the disease progression (Naughton et al., 1995; Esler et al., 2001; Mansfield et al., 2003). Human and experimental animal studies revealed that both heart failure and systemic arterial hypertension are associated with lower brain *P*O_2_ even when arterial *P*O_2_ is within the normal physiological range (Rifai, Winters, Friedman, & Silver, 2012; Marina et al., 2015; Turlejski, Humoud, Desai, Smith, & Marina, 2016; Hosford, Millar, Ramage, & Marina, 2017). The mechanisms underlying compromised brain oxygenation in these conditions are complex and remain poorly understood (Cates, Steed, Abdala, Langton, & Paton, 2011; Cates, Dickinson, Hart, & Paton, 2012; Marina et al., 2015; Marina, Teschemacher, Kasparov, & Gourine, 2016), however, brainstem parenchymal hypoxia might contribute to sustained excitation of the pre‐sympathetic circuits via the actions of ATP and lactate released by astrocytes at low tissue *P*O_2_ (Marina et al., 2013; Marina et al., 2015). To test this hypothesis, two studies in rats used viral gene targeting of pre‐sympathetic regions of the brainstem to overexpress a potent ectonucleotidase—transmembrane prostatic acid phosphatase. Activity of this enzyme effectively prevents vesicular accumulation of ATP (Wells et al., 2015) and facilitates degradation of extracellular ATP (Marina et al., 2013). Blockade of ATP‐mediated signaling within the pre‐sympathetic brainstem regions slowed the progression of cardiac remodeling in animals with myocardial infarction‐induced heart failure (Marina et al., 2013) and reduced systemic arterial blood pressure in spontaneously hypertensive rats (Marina et al., 2015). Subsequent studies provided further evidence of brainstem tissue hypoxia and astrogliosis in hypertensive rats (Turlejski et al., 2016). Together these data support the hypothesis that sustained astroglial activation in conditions of brainstem tissue hypoxia might be responsible for maintaining heightened sympathetic drive that contributes to the development and progression of cardiovascular disease (Marina et al., 2013; Marina et al., 2015; Marina et al., 2016).

Finally, there is evidence that brain tissue *P*O_2_ is the key metabolic factor that determines the direction of cerebral arteriole response (constriction at high *P*O_2_ and dilatation at physiological/low *P*O_2_) that follow astroglial [Ca^2+^]_i_ elevations (Gordon, Choi, Rungta, Ellis‐Davies, & MacVicar, 2008). Since Ca^2+^‐dependent release of vasoactive substances by astrocytes can alter cerebral blood flow (Attwell et al., 2010; Mishra et al., 2016; Bazargani & Attwell, 2016), the mechanism of direct oxygen sensing by astrocytes (Angelova et al., 2015) may be important for the regulation of cerebral microcirculation in conditions of increased local oxygen demand or regional brain tissue hypoxia. While this hypothesis awaits experimental scrutiny, the available data strongly suggest that detection of hypoxia by brainstem astrocytes stimulates the networks of the respiratory and pre‐sympathetic neurons and contributes to the development of the ventilatory and cardiovascular responses which ensure appropriate oxygenation and delivery of the arterial blood.

### 3.2 Sensing glucose

Glucose is an important source of energy and a substrate for many biochemical reactions. Blood glucose level fluctuates between ∼70 and 100 mg dl^−1^ throughout a 24‐hr period in fasting conditions and may increase up to 140 mg dl^−1^ within the first 2 hr after ingestion of a meal. Intricate neural and hormonal control mechanisms operate to maintain blood glucose level within a physiological range and to ensure that the metabolic demands of all tissues of the body, and of the brain in particular, are satisfied.

Diabetic patients treated with insulin and sulphonylureas are at increased risk of acute hypoglycemia. Hypoglycemia can have profound deleterious effects on the neuronal function, leading to permanent brain damage and even death (Frier, 2014). Sustained elevations in plasma glucose can also have various adverse effects on vital organs, including the brain. Therefore, physiological glucose sensing is critically important for homeostasis which is ensured via recruitment of the hormonal (insulin and glucagon secretion), autonomic (liver glucose production) and behavioral (feeding initiation and termination) mechanisms.

There is evidence that brainstem astrocytes may function as CNS glucose sensors. Mice with genetic deletion of the glucose transporter type 2 (GLUT2) were not able to respond to systemic hypoglycemia with increased glucagon secretion (Marty et al., 2005). However, the capacity to release glucagon in response to hypoglycemia was restored by selective re‐expression of GLUT2 in the brainstem glial cells, but not in neurons (Marty et al., 2005). An earlier study reported that hypoglycemia‐induced activation of neurons in the hypothalamus and the brainstem was blocked in conditions when astroglial glutamate metabolism was compromised by application of glutamine synthetase inhibitor methionine sulfoximine (Young, Baker, & Montes, 2000). Together, these data suggest that detection of hypoglycemia by the brain may require metabolic coupling and signaling between astrocytes and neurons. Consistent with this hypothesis, astrocytes have been shown to control (via release of lactate) the activities of hypothalamic orexin neurons which promote arousal, stimulate food intake and hepatic glucose production (Parsons & Hirasawa, 2010). However, the cellular and molecular mechanisms underlying glucose sensitivity of astrocytes remain to be determined.

Recently, Garcia‐Caceres and colleagues (2016) reported data suggesting that astroglial insulin signaling modulates hypothalamic glucose sensing and systemic glucose metabolism. The authors demonstrated that ablation of insulin receptors in hypothalamic astrocytes reduced glucose‐induced activation of pro‐opio‐melanocortin neurons and impaired physiological responses to changes in glucose availability. Following systemic glucose administration, cerebrospinal fluid accumulation of glucose and insulin were found to be reduced in mice lacking astroglial insulin receptors. The authors concluded that brain glucose sensing and, therefore, systemic glucose metabolism are controlled, at least in part, by insulin acting at hypothalamic astrocytes. Moreover, the data reported by Garcia‐Caceres et al. (2016) also suggested that astroglial insulin receptors play an important role in modulating glucose transfer across the blood–brain barrier.

### 3.3 Sensing carbon dioxide and pH

Metabolic homeostasis also requires effective elimination of waste products. Carbon dioxide is generated in proportion to the metabolic rate and the amount of metabolic substrates utilized. At rest, our body produces ∼12 mmol kg^−1^ h^−1^ of CO_2_; most of which is removed with expired air through the process of alveolar ventilation. The partial pressure of CO_2_ (*P*CO_2_) in the arterial blood is directly proportional to the rate of CO_2_ production and inversely proportional to the rate of CO_2_ elimination by the respiratory system. Increased CO_2_ production and/or impaired CO_2_ elimination facilitate generation of hydrogen ions (respiratory acidosis), a condition that needs to be rapidly corrected by adaptive changes in the ventilatory and cardiovascular activities in order to ensure effective CO_2_ removal.

It is generally believed that changes in the arterial and brain *P*CO_2_/pH are monitored by specialized pH‐sensitive neurons residing in the brainstem (Loeschcke, 1982). Current models of central respiratory CO_2_ chemosensitivity (the mechanism that adjusts breathing in accordance with changes in brainstem parenchymal *P*CO_2_/pH) are focused on a group of pH‐sensitive neurons of the retrotrapezoid nucleus (RTN) located near the ventral surface of the brainstem (Guyenet, & Mulkey, 2010). This view is supported by the results of the experimental studies which demonstrated that the permanent loss or acute silencing of RTN neurons abolishes or significantly reduces ventilatory CO_2_ sensitivity (Dubreuil et al., 2008; Guyenet et al., 2009; Guyenet & Mulkey, 2010; Marina et al., 2010; Ramanantsoa et al., 2011). Although other notable groups of brainstem neurons, including 5‐HT neurons of the raphe nuclei, are intrinsically chemosensitive (Teran, Massey, & Richerson, 2014) and contribute to the development of the ventilatory response to CO_2_ (Ray et al., 2011), the current prevailing view is that pH‐sensitive RTN neurons play the key role (Guyenet et al., 2016).

**Figure 3:**
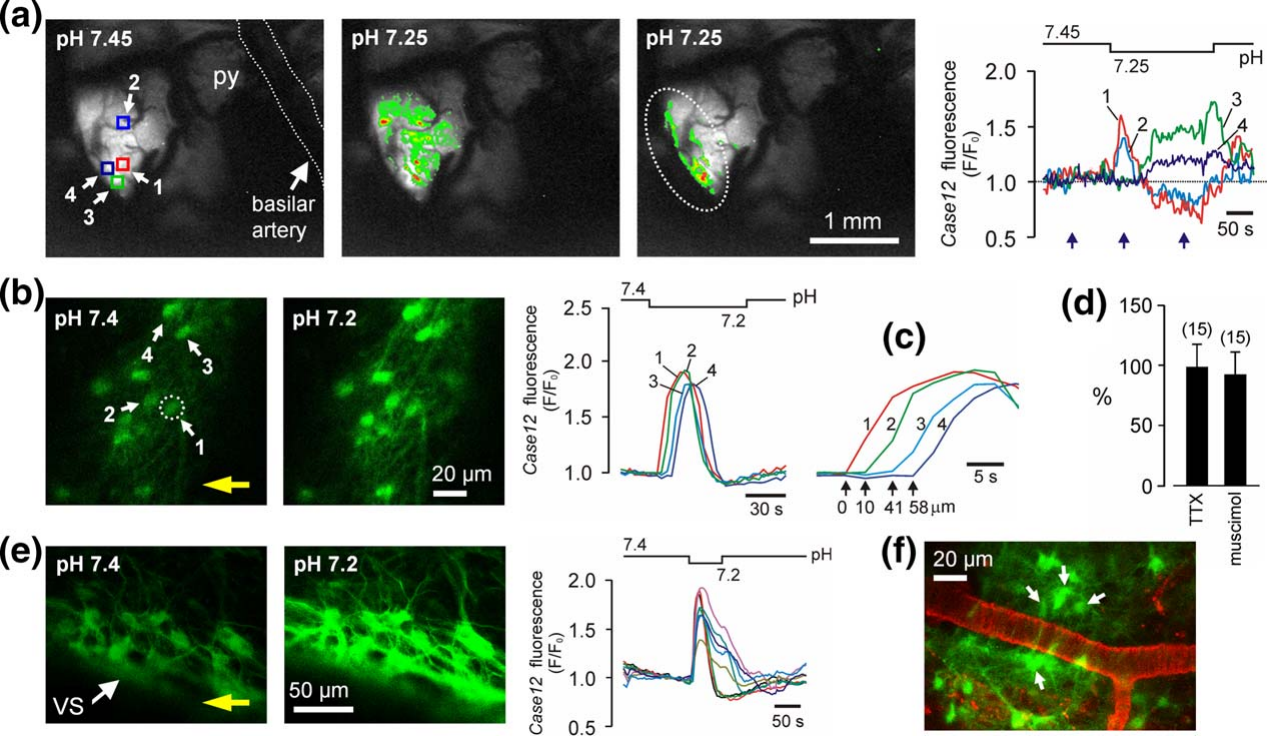
Astrocytes residing near the ventral surface of the brainstem are exquisitely pH‐sensitive. (a) *In vivo* imaging of pH‐evoked astrocytic [Ca^2+^]_i_ responses in the ventrolateral area of the brainstem surface transduced with AVV‐sGFAP‐*Case12* in an anesthetized adult rat. Exposed ventral brainstem surface was continuously superfused with HEPES‐buffered solutions of variable pH and bulk [Ca^2+^]_i_ responses to a 0.2 unit pH decrease were recorded. A large area of the brainstem was imaged at low magnification, therefore, regions of interest in this case encompass multiple astrocytes which cannot be individually resolved under these imaging conditions. Right traces: changes in astrocytic [Ca^2+^]_i_ in response to a decrease in pH. Pseudocolored images (left) were taken at times indicated by blue arrows. Squares indicate regions of interest. The pH bar shows when the solution with lower pH is reaching and starts leaving the exposed ventral surface of the brainstem. Dashed line outlines approximate boundary of the chemosensitive retrotrapezoid nucleus. py—pyramidal tract. (b) Astrocytes identified by *Case12* fluorescence in a horizontal slice from an adult rat in which the ventral brainstem was transduced with AVV‐sGFAP‐*Case12*. Acidification induces rapid increases in [Ca^2+^]_i_ as determined by changes in *Case12* fluorescence. Two fluorescent images obtained before and at the peak of [Ca^2+^]_i_ response. Circle indicates an astrocyte responding first to pH change in the field of view. Yellow arrow shows the direction of the flow in the chamber. (c) Zoomed in Ca^2+^ transients to emphasize the latency differences between responses of individual astrocytes shown in (b). (d) No effect of TTX or muscimol on acidification‐induced [Ca^2+^]_i_ responses in brainstem astrocytes expressed as percentage of the peak initial response. Numbers of individual astrocytes sampled from 3–5 separate experiments are given in brackets. (e) Acidification‐evoked [Ca^2+^]_i_ responses in astrocytes of organotypic brainstem slice transduced with AVV‐sGFAP‐*Case12*. (f) Brainstem vasculature visualized with lectin in a horizontal slice prepared from an AVV‐sGFAP‐Case12–transduced rat. Arrows point at pH‐responsive astrocytes. Reproduced from Gourine et al. (2010) with permission from AAAS

However, there is evidence that pH‐sensitivity of RTN neurons is, to a large extent, indirect and mediated by the responses triggered by the chemosensory stimuli in the neighboring astrocytes (Gourine et al., 2010). Experiments conducted in anesthetized and mechanically ventilated rats demonstrated that astrocytes residing near the ventral surface of the brainstem (within the RTN region) respond to decreases in pH with robust elevations in intracellular [Ca^2+^] (Gourine et al., 2010; Figure 3). This triggers Ca^2+^‐dependent vesicular release of ATP (Gourine et al., 2010; Kasymov et al., 2013) which propagates Ca^2+^ excitation among neighbouring astrocytes, activates RTN neurons (Gourine et al., 2010) as well as respiratory neurons that constitute other functional divisions of the ventral respiratory column (Gourine et al., 2003).

The mechanisms linking the detection of *P*CO_2_/[H^+^] increases with [Ca^2+^]_i_ responses in brainstem astrocytes are dependent on the activities of certain membrane transporters (Turovsky et al., 2016). Astroglial [Ca^2+^]_i_ responses triggered by CO_2_‐induced acidification (respiratory acidosis) were found to be preceded by Na^+^ entry, reduced by inhibition of the Na^+^/ ${\text{HCO}}_{3}^{-}$ cotransport (NBC) or Na^+^/Ca^2+^ exchange (NCX) and abolished in the absence of extracellular Na^+^. Acidification‐induced Ca^2+^ responses were also dramatically reduced in brainstem astrocytes of mice deficient in the electrogenic Na^+^/ ${\text{HCO}}_{3}^{-}$ cotransporter NBCe1 (Turovsky et al., 2016). Thus, coupled NBC and NCX activities appear to underlie functional pH‐sensitivity of brainstem astrocytes leading to the increases in intracellular [Ca^2+^] (Figure 1).

Ventral brainstem astrocytes also possess the mechanism of direct CO_2_ sensing (Huckstepp et al., 2010) which operates via modulation of connexin‐26 hemichannel opening (Meigh et al., 2013). The CO_2_‐dependent hemichannel‐mediated release of ATP operates independently of astroglial pH sensing mechanism(s), since pharmacological agents which block functional connexin hemichannels have little effect on acidification‐induced Ca^2+^ responses in brainstem astrocytes (Gourine et al., 2010).

Unlike direct oxygen sensing which is a ubiquitous feature shared between astrocytes residing in different CNS regions (Angelova et al., 2015), CO_2_/pH sensitivity appears to be a unique feature of specialized astrocytes which populate the brainstem areas at and in a close proximity to its ventral surface (Gourine et al., 2010; Kasymov et al., 2013; Turovsky et al., 2016). This was demonstrated in a comparative study where only brainstem astrocytes were found to display responses to pH challenges (Kasymov et al., 2013). Transcriptome analysis identified NBCe1 expression to be markedly higher in the brainstem astrocytes compared with that in cortical astrocytes (Turovsky et al., 2016). Brainstem astrocytes also showed significantly higher expression of another notable astroglial gene, KCNJ10 (Turovsky et al., 2016), which encodes the K_IR_4.1 subunit of inwardly rectifying K^+^ channel. In mice, astrocyte‐specific conditional deletion of K_IR_4.1 was reported to impair ventilatory CO_2_ chemosensitivity (Hawkins et al., 2014).

CO_2_‐induced hemichannel‐mediated and/or acidification‐induced vesicular release of ATP by brainstem astrocytes contributes to the homeostatic regulation of brain pH by triggering adaptive changes in the brainstem respiratory network activity and, therefore, lung ventilation, facilitating CO_2_ removal. Systemic hypercapnia (induced by increases in inspired CO_2_) in anesthetized and mechanically ventilated rats was reported to trigger release of ATP from the ventral brainstem surface (Gourine et al., 2005; Huckstepp, Llaudet, & Gourine, 2016). This CO_2_‐induced release of ATP was dependent upon the structural integrity of the subpial astrocyte layer and occurred prior to the increases in central respiratory drive (Gourine et al., 2005; Huckstepp et al., 2010). Furthermore, CO_2_‐induced ventilatory responses were significantly reduced following blockade of ATP receptors at the sites of release (Gourine et al., 2005). The effect of CO_2_ on breathing was mimicked by application of ATP to the brainstem sites of release as well as by optogenetic stimulation of brainstem astrocytes (Gourine et al., 2005; Gourine et al., 2010; Figueiredo et al., 2011).

Impaired astroglial mechanisms may contribute to the development of abnormal breathing patterns observed in some prototypical neurological disorders. In humans, mutations of the transcriptional regulator methyl‐CpG‐binding protein 2 (*MeCP2*) gene lead to a neurodevelopmental disorder called Rett syndrome, which is characterized by irregular breathing pattern and blood gas instability (Southall et al., 1988; Viemari et al., 2005; Ramirez, Ward, & Neul, 2013). *MeCP2* is highly expressed in astrocytes (Yasui et al., 2013) and loss of *MeCP2* leads to astroglial dysfunction (Okabe et al., 2012). In a mouse model of Rett syndrome (global MeCP2 gene knockout), sensitivity of brainstem astrocytes to changes in *P*CO_2_/pH is markedly reduced (Turovsky, Karagiannis, Abdala, & Gourine, 2015) and ventilatory CO_2_ sensitivity is severely impaired (Bissonnette, Schaevitz, Knopp, & Zhou, 2014). Moreover, it was reported that in mice, conditional astrocyte‐specific deletion of *MeCP2* is sufficient to dramatically impair CO_2_‐induced ventilatory response (Garg, Lioy, Knopp, & Bissonnette, 2015). Remarkably, in global MeCP2 gene knockout mice, selective re‐expression of *MeCP2* in astrocytes rescues the normal respiratory pattern (Lioy et al., 2011). These data indicate that the brainstem networks of the respiratory neurons, including chemosensitive RTN neurons, are not able to mount an appropriate ventilatory response to CO_2_ when astroglial function and pH‐sensitivity are compromised, supporting the idea of a critical role played by astroglial pH‐sensitivity in the CNS mechanisms which transmit changes in brain parenchymal *P*CO_2_/pH into a modified pattern of breathing. It remains to be determined whether astroglial dysfunction may also contribute to the expression of altered breathing patterns (e.g., central sleep apnoea) observed in some other pathological conditions.

Finally, a recent study has suggested that astrocytes may also mediate cerebrovascular responses to CO_2_. Howarth and colleagues (2017) reported that in anesthetized mice, increases in the level of inspired CO_2_ trigger [Ca^2+^]_i_ responses in cortical astrocytes, which in turn may evoke cerebral vessel dilations via stimulation of COX‐1 activity followed by PgE_2_ release and its action on cerebrovascular smooth muscle cells (Howarth et al., 2017).

## 4 ASTROCYTES AS CNS SENSORS OF METABOLIC ENDOCRINE SIGNALS

Metabolic homeostasis is ensured by coordinated adaptive physiological and behavioral responses to a multitude of endogenous and environmental factors and most importantly by the availability of nutrients. The control of food intake in particular is generally believed to depend on the ability of specialized neurons located in the hypothalamus and the brainstem to detect and integrate various humoral and afferent neuronal signals which provide information about the nutritional state and energy demands of the organism (Caron & Richard, 2017). Considering that astrocytes function as CNS metabolic sensors we next explored whether these glial cells are sensitive to endocrine metabolic signals released from the gut and the adipose tissue. The data reported below were obtained using standard experimental models and protocols described in detail in our previous publications (Gourine et al., 2010; Kasymov et al., 2013; Angelova et al., 2015; Turovsky et al., 2015; Turovsky et al., 2016).

### 4.1 Ghrelin

Ghrelin (also known as growth hormone‐releasing peptide) is the only circulating peptide known to stimulate appetite and increase food intake. Ghrelin is mainly produced and released by oxyntic glands of the gastric fundus and its CNS actions increase food intake, produce weight gain, and promote adiposity via increased production of orexigenic neuropeptides such as neuropeptide Y and Agouti‐related peptide (AGRP) by the neurons of the arcuate nucleus of the hypothalamus (Wren et al., 2001; Greenman et al., 2004). There is evidence that the effect of ghrelin on food intake is suppressed by activation of astrocytes which inhibit hypothalamic AGRP‐producing neurons via the release of ATP/adenosine acting at A1 receptors (Yang, Qi, & Yang, 2015).

**Figure 4:**
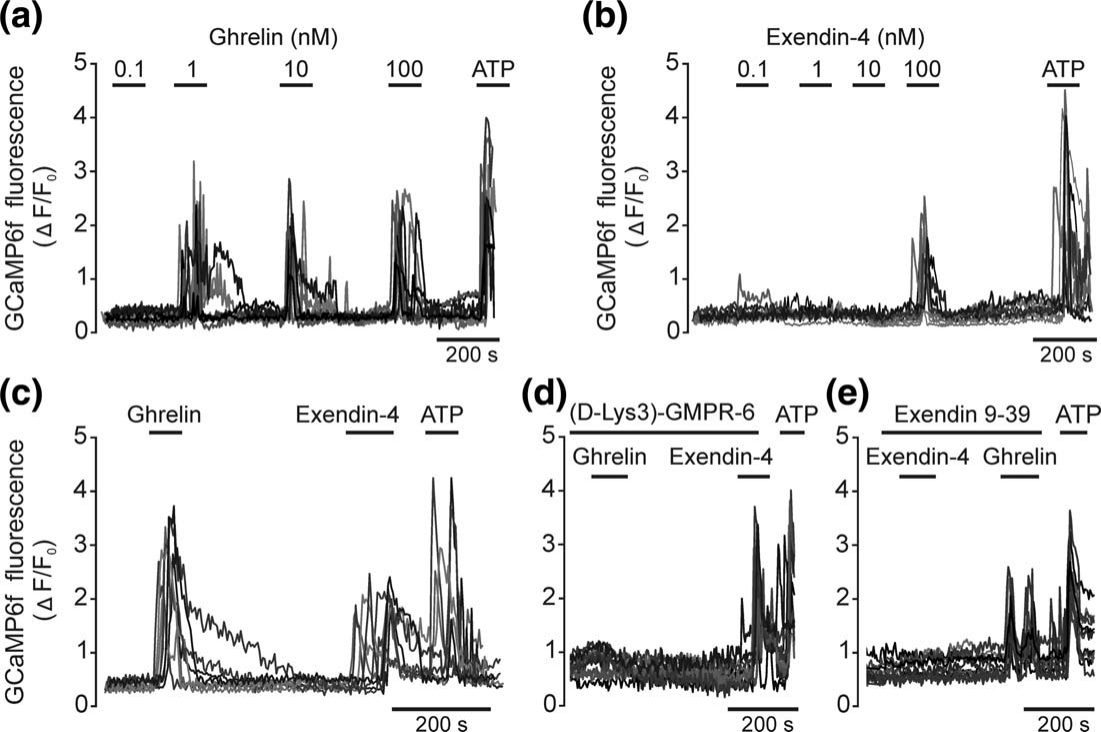
Brainstem astrocytes are sensitive to ghrelin and GLP‐1 receptor activation. (a) Representative example of astroglial [Ca^2+^]_i_ responses induced by ghrelin applied in increasing concentrations. Traces depict responses of nine individual astrocytes transduced to express Ca^2+^ indicator GCaMP6f in culture. Here and in the other examples [Ca^2+^]_i_ responses triggered by ATP (10 µM) applied at the end of the recordings confirm cell viability. (b) Representative example of astroglial [Ca^2+^]_i_ responses induced by GLP‐1 receptor agonist Exendin‐4 applied in increasing concentrations. Traces depict responses of nine individual astrocytes in culture. (c) Representative example of [Ca^2+^]_i_ responses of the same brainstem astrocytes induced by successive applications of ghrelin (10 nM) and Exendin‐4 (100 nM). Traces depict responses of eight individual astrocytes. (d) Representative recording illustrating the effect of GHSR1 antagonist [(D‐Lys3)‐GMPR6] (100 µM) on [Ca^2+^]_i_ responses of brainstem astrocytes induced by ghrelin (100 nM) and Exendin‐4 (100 nM). Individual traces of [Ca^2+^]_i_ responses in 13 astrocytes are shown. (e) Representative recording illustrating the effect of GLP‐1 receptor antagonist Exendin 9–39 (1 µM) on [Ca^2+^]_i_ responses of brainstem astrocytes induced by Exendin‐4 (100 nM) and ghrelin (100 nM). Individual traces of [Ca^2+^]_i_ changes in 11 astrocytes are shown

To determine the effect of ghrelin on astrocytes we used dissociated neuroglial cultures transduced to express a genetically encoded Ca^2+^ indicator GCaMP6f. Astrocytes were transduced using an adeno‐associated viral vector designed to drive the expression of GCaMP6f under the transcriptional control of the GFAP promoter (Jiang, Haustein, Sofroniew, & Khakh, 2014). The majority of astrocytes expressing GCaMP6f responded to application of a prototypical glial signaling molecule ATP (10 µM) with elevations in [Ca^2+^]_i_ (Figure 4a–e). Ghrelin triggered robust [Ca^2+^]_i_ responses in ∼30% of brainstem astrocytes that were activated by ATP (Figure 4a). Strong [Ca^2+^]_i_ responses in astrocytes were induced following application of ghrelin in concentrations as little as 1 nM (Figure 4a). Ghrelin‐induced [Ca^2+^]_i_ responses in astrocytes were effectively blocked by ghrelin receptor (GHSR1a) antagonist [(D‐Lys3)‐GMPR‐6] (Figure 4d).

These data suggest that astrocytes (at least in culture) express functional ghrelin receptors and respond to physiological concentrations of ghrelin with elevations in intracellular [Ca^2+^]. It remains to be determined whether astrocytes *in situ* are sensitive to ghrelin and may ultimately mediate or modulate the effects of ghrelin on neighboring neurons.

### 4.2 Glucagon‐like peptide (GLP‐1)

GLP‐1 is one of many hormones known to induce satiety. GLP‐1 belongs to the family of incretin peptides that are produced by the intestinal epithelial L‐cells (Tian & Jin, 2016). Actions of GLP‐1 include stimulation of glucose‐dependent insulin secretion, inhibition of glucagon secretion and stimulation of somatostatin secretion (Tian & Jin, 2016). GLP‐1 also delays gastric emptying, inhibits gastrointestinal motility, and contributes to the physiological control of feeding behaviour (Holst, 2007). GLP‐1 receptors (GLP‐1R) are expressed in the brain regions involved in the control of energy metabolism, such as mediobasal hypothalamus and the caudal brainstem (Cork et al., 2015) and central actions of GLP‐1 reduce food intake (Tang‐Christensen et al., 1996; Turton et al., 1996). There is recent evidence suggesting that astrocytes may mediate the effects of central GLP‐1R activation on feeding behavior. Results of the experiments conducted in rat brainstem slices showed that astrocytes residing in the dorsal brainstem respond to GLP‐1R activation with elevations in [Ca^2+^]_i_ (Reiner et al., 2016). Moreover, poisoning of dorsal brainstem astrocytes with fluorocitrate attenuated the effect of GLP‐1R activation on food intake when GLP‐1 analog Exendin‐4 (Ex‐4) was administered into the same brainstem site (Reiner et al., 2016). However, these data should be interpreted with some caution since fluorocitrate is not a selective “glial toxin” and depending on the concentration and the experimental conditions could potentially inhibit tricarboxylic acid cycle of all cells.

Experiments conducted using cultures of brainstem astrocytes transduced to express GCaMP6f revealed that relatively high concentrations of GLP‐1R agonist Ex‐4 are needed to elicit [Ca^2+^]_i_ responses (Figure 4b). The threshold dose of Ex‐4 required to trigger robust [Ca^2+^]_i_ elevations in cultured astrocytes was found to be 100 nM (Figure 4b). [Ca^2+^]_i_ responses to GLP‐1R activation were observed in ∼30% of astrocytes that responded to the application of ATP. Ex‐4‐induced [Ca^2+^]_i_ responses in astrocytes were abolished in the presence of the GLP‐1R antagonist Exendin 9–39 (Figure 4e). Interestingly, astrocytes that displayed [Ca^2+^]_i_ responses to ghrelin were also sensitive to GLP‐1R activation (Figure 4c). Blockade of GLP‐1R had no effect on [Ca^2+^]_i_ responses triggered by ghrelin, while blockade of GHSR1a receptors had no effect on Ex‐4‐induced [Ca^2+^]_i_ transients (Figure 4d,e). These data suggest that individual brainstem astrocytes are able to respond to various endocrine signals with apparently opposing central physiological actions.

There is evidence that the central effects of GLP‐1 analogs are associated with excitation of pre‐sympathetic C1 neurons, increases in central sympathetic drive, systemic arterial blood pressure and heart rate (Yamamoto et al., 2002). Although, these effects are likely to be attributed to direct activation of neuronal GLP‐1Rs, the data reported and discussed above suggest that brainstem astrocytes may contribute to GLP‐1‐induced sympathoexcitation via Ca^2+^‐dependent release of signaling molecules that activate pre‐sympathetic circuits (e.g., ATP). This hypothesis can be tested in the future by recording the sympathetic and cardiovascular effects of GLP‐1R activation within the sympathoexcitatory brainstem regions in conditions when astroglial signaling pathways are blocked using molecular approaches.

### 4.3 Leptin

Leptin is a hormone produced mainly by the white and brown adipose tissue that plays an important role in the control of energy metabolism. Leptin crosses the blood–brain barrier through a saturable transport system (Banks, Kastin, Huang, Jaspan, & Maness, 1996) and induces receptor (ObR)‐mediated inhibition of the release of orexigenic peptides (neuropeptide Y and AGRP) produced by the neurons of the hypothalamic arcuate nucleus (Elias et al., 1999; van den Top, Lee, Whyment, Blanks, & Spanswick, 2004). Leptin also stimulates the release of anorectic peptides (e.g., pro‐opio melanocortin; Elias et al., 1999; Cowley et al., 2001). These actions of leptin inhibit appetite and increase energy expenditure.

Previously the central effects of leptin were thought to be primarily mediated via its direct actions on hypothalamic neurons and endothelial cells but recent evidence suggests that some of these effects are mediated by astrocytes. Astrocytes express several ObR splice variants and leptin application has been shown to trigger strong and sustained [Ca^2+^]_i_ responses in primary astrocytes from mouse hypothalamus (Hsuchou, Pan, Barnes, & Kastin, 2009). Mice with genetic‐ and diet‐induced obesity show an upregulation of ObR expression in hypothalamic astrocytes and a concomitant downregulation of ObR expression in neurons (Hsuchou et al., 2009; Pan et al., 2008). Another study reported enhanced leptin uptake by hypothalamic neurons in the presence of fluorocitrate, suggesting that astrocytes may play a certain role in the distribution of this hormone among different cellular compartments (Pan et al., 2011).

Several studies conducted in rats reported that direct administration of leptin to the pre‐sympathetic regions of the brainstem triggers profound and sustained increases in central sympathetic drive and systemic arterial blood pressure (Haynes, Morgan, Walsh, Mark, & Sivitz, 1997; Barnes & McDougal, 2014). Moreover, leptin actions in the brainstem were found to enhance the baseline respiratory activity and ventilatory sensitivity to CO_2_ in leptin deficient mice (ob/ob; Bassi et al., 2014). These effects of leptin on the respiratory and sympathetic activities mirror the effects of astroglial activation. However, it remained unclear whether the cardiorespiratory effects of leptin result from its direct actions on the respiratory and pre‐sympathetic neuronal circuits or secondary to the responses elicited by this hormone in neighboring astrocytes.

**Figure 5:**
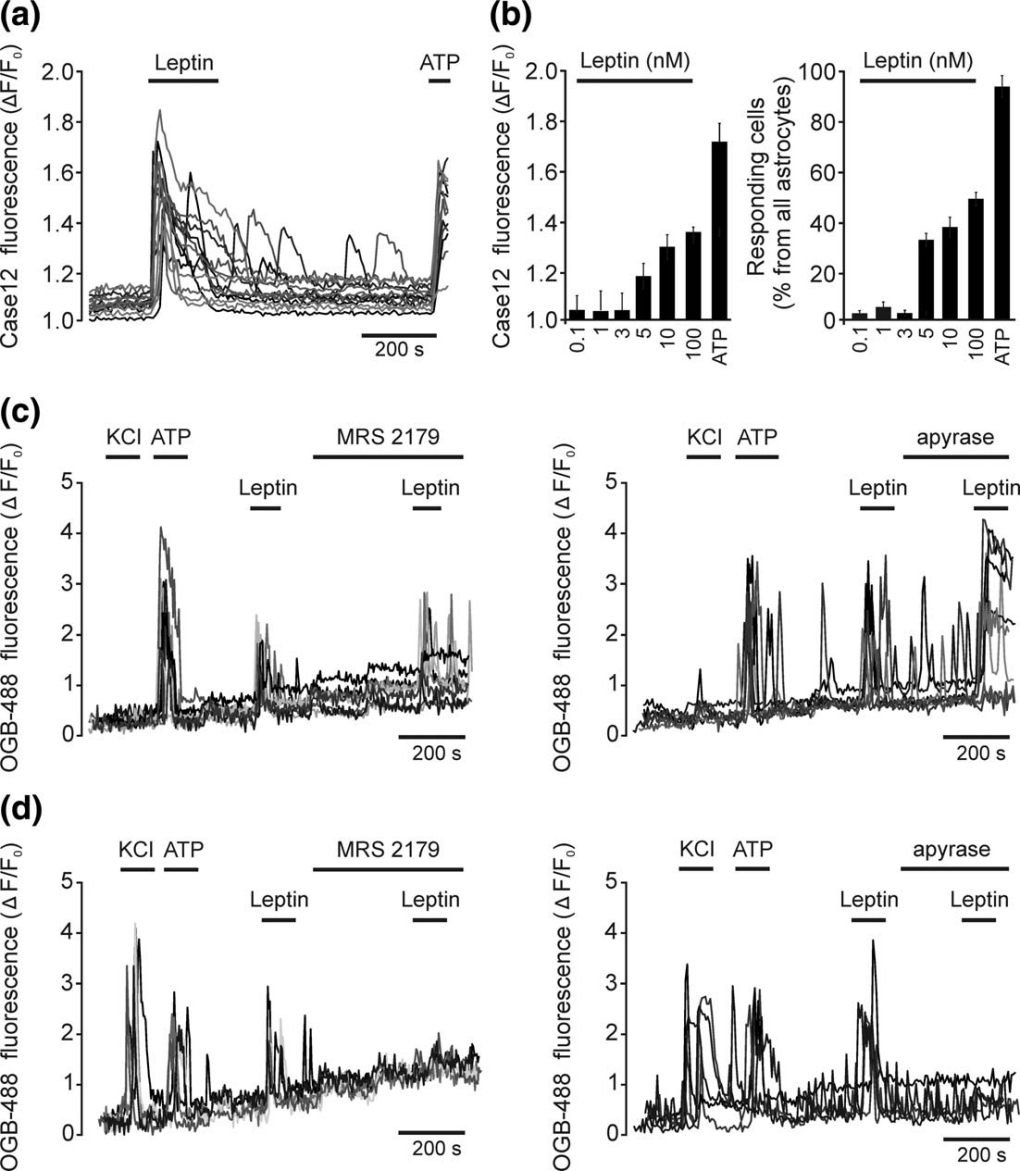
Leptin‐induced [Ca^2+^]_i_ responses in brainstem astrocytes and neurons. (a) Representative example of [Ca^2+^]_i_ responses induced by leptin (10 nM) in cultured astrocytes. Traces depict responses of 20 individual astrocytes transduced to express a Ca^2+^ indicator *Case12*. (b) Summary data illustrating peak amplitudes of astroglial [Ca^2+^]_i_ responses and proportion of astrocytes responding with [Ca^2+^]_i_ elevations to leptin applied in increasing concentrations. (c) Representative examples of [Ca^2+^]_i_ responses induced by leptin (10 nM) in astrocytes (identified by labeling with sulforhodamine 101) in the absence and presence of an ATP receptor blocker MRS2179 (10 µM) or ATP‐hydrolyzing enzyme apyrase (50 U ml^−1^). Traces depict responses of 8 (left) and 8 (right) individual astrocytes loaded with oregon green‐488 BAPTA‐1 AM (OGB‐488) in organotypic slices of the rat brainstem. (d) Representative examples of [Ca^2+^]_i_ responses induced by leptin (10 nM) in neurons (identified by lack of sulforhodamine 101 labeling and robust [Ca^2+^]_i_ responses to KCl) in the absence and presence of MRS2179 (10 µM) or apyrase (50 U ml^−1^). Traces depict responses of 5 (left) and 5 (right) individual astrocytes loaded with oregon green‐488 BAPTA‐1 AM (OGB‐488) in organotypic slices of the rat brainstem

We evaluated the effect of leptin on [Ca^2+^]_i_ in cultured brainstem astrocytes transduced to express the genetically encoded Ca^2+^ indicator *Case12* under the control of the GFAP promoter (Gourine et al., 2010). Leptin evoked robust [Ca^2+^]_i_ responses in ∼40% of brainstem astrocytes that responded to ATP application (Figure 5a,b). The threshold dose of leptin required to trigger [Ca^2+^]_i_ elevations in these astrocytes was found to be 5 nM (Figure 5b).

To test whether changes in the neuronal activity induced by leptin might be secondary to astroglial activation and mediated by the release and actions of gliotransmitters, we next determined the effect of leptin on [Ca^2+^]_i_ in astrocytes and neurons recorded in organotypic brainstem slices (cut at the “pre‐sympathetic level”) bulk‐loaded with the Ca^2+^ indicator Oregon Green‐488 BAPTA‐1 AM (OGB‐488). Astrocytes were identified by labeling with sulforhodamine 101 (SR101) as described previously (Turovsky et al., 2015). Cells labeled with SR101 displayed robust [Ca^2+^]_i_ responses to ATP application and were insensitive to KCl (Figure 5c), indicating that these cells are astrocytes. Leptin‐induced [Ca^2+^]_i_ responses in astrocytes were unaffected in the presence of P2Y receptor antagonist MRS2179 or ATP‐hydrolyzing enzyme apyrase (Figure 5c). In contrast, leptin‐induced [Ca^2+^]_i_ responses in brainstem neurons (identified by lack of SR101 labeling, and robust [Ca^2+^]_i_ responses to KCl) were abolished by either MRS2179 or apyrase (Figure 5d). Although the phenotype of the neurons recorded in these experiments was not characterized, these results suggest that the CNS behavioral and physiological effects of leptin may involve recruitment of astroglial signaling pathways, release of gliotransmitter ATP and activation of the neuronal purinergic receptors.

Together, these data suggest that astrocytes residing in brain regions involved in the control of energy metabolism (hypothalamus and the brainstem) are sensitive to key hormonal factors whose central actions provide important information about the nutritional state and energy demands of the organism. It remains to be determined whether these sensitivities are exclusive features of astrocytes residing in brain areas involved in metabolic control. It also remains to be determined whether chronic exposure to the elevated levels of these hormones may alter astroglial function and signaling mechanisms. This may have a significant impact on the control of feeding behavior and cardiorespiratory homeostasis and ultimately contribute to the pathogenesis of metabolic and/or cardiovascular disease.

## 5 SUMMARY

There is growing evidence to suggest that astrocytes actively monitor CNS metabolic milieu and contribute to the development of adaptive physiological respiratory, cardiovascular and behavioral responses which maintain metabolic homeostasis. Anatomical and functional features of astrocytes allow them to detect and respond to changes in the brain parenchymal levels of metabolic substrates (O_2_ and glucose), metabolic by products (CO_2_), and hormonal metabolic factors involved in the CNS mechanisms controlling food intake and energy balance. In the brainstem, astrocytes modulate the activities of the neuronal circuits responsible for the generation of the respiratory and autonomic rhythms and the development of the adaptive changes in breathing and sympathetic nerve activity in conditions of increased metabolic demand. The key signaling molecule which mediates communication between astrocytes and the brainstem cardiorespiratory networks appears to be ATP, although other gliotransmitters (e.g., lactate) may also play a role. Furthermore, there is evidence that altered astroglial function may contribute to the pathogenesis of disparate respiratory and cardiovascular disorders such as Rett syndrome, heart failure and systemic arterial hypertension.
